# Identification of reference genes for qPCR analysis during hASC long culture maintenance

**DOI:** 10.1371/journal.pone.0170918

**Published:** 2017-02-09

**Authors:** Silvia Palombella, Cristina Pirrone, Mario Cherubino, Luigi Valdatta, Giovanni Bernardini, Rosalba Gornati

**Affiliations:** 1 Department of Biotechnology and Life Sciences, University of Insubria, Varese, Italy; 2 The Protein Factory Research Center, Politecnico di Milano, ICRM-CNR Milano and University of Insubria, Milano, Italy; Morehouse School of Medicine, UNITED STATES

## Abstract

Up to now quantitative PCR based assay is the most common method for characterizing or confirming gene expression patterns and comparing mRNA levels in different sample populations. Since this technique is relative easy and low cost compared to other methods of characterization, e.g. flow cytometry, we used it to typify human adipose-derived stem cells (hASCs). hASCs possess several characteristics that make them attractive for scientific research and clinical applications. Accurate normalization of gene expression relies on good selection of reference genes and the best way to choose them appropriately is to follow the common rule of the “Best 3”, at least three reference genes, three different validation software and three sample replicates. Analysis was performed on hASCs cultivated until the eleventh cell confluence using twelve candidate reference genes, initially selected from literature, whose stability was evaluated by the algorithms NormFinder, BestKeeper, RefFinder and IdealRef, a home-made version of GeNorm. The best gene panel (*RPL13A*, *RPS18*, *GAPDH*, *B2M*, *PPIA* and *ACTB*), determined in one patient by IdealRef calculation, was then investigated in other four donors. Although patients demonstrated a certain gene expression variability, we can assert that *ACTB* is the most unreliable gene whereas ribosomal proteins (*RPL13A* and *RPS18*) show minor inconstancy in their mRNA expression. This work underlines the importance of validating reference genes before conducting each experiment and proposes a free software as alternative to those existing.

## Introduction

Stem cells are unspecialized cells that possess three basic characteristics: self-renewal, long life and ability to differentiate into various cell lines. Human adult somatic stem cells are present throughout all the body including adipose tissue [1,2]. Later studies showed the potential ability of adipose stem cells (hASCs) to differentiate towards different cell lineages [3–8] and therefore, it was recently suggested that the hASCs can be pluripotent. hASCs are very attractive for scientific research and clinical applications as they can be easily extracted and cultivated *in vitro*, have a greater differentiation potential than other mesenchymal stem cells and are optimal for allograft as they have low immunogenicity. Moreover, the use of adult stem cells overcomes ethical problems connected with embryonic stem cells.

Unfortunately, despite their great potential there is a lack of standardized methods concerning the surface marker characterization [9]. This evaluation is usually performed by flow cytometry but quantitative PCR (qPCR) could be used as an alternative method as it is relative easy to perform and cheaper than other techniques. Nowadays this method is widely used for the evaluation of gene expression patterns and for comparing mRNA levels in different population samples [10,11].

When using relative quantification in qPCR, the basic assumption is the selection of proper reference genes (RGs) in order to adequately normalize the expression of target genes, thus correcting sample-to-sample variations. A good RG is characterized by a permanent and unchanging expression in each of tested samples, despite the impact of experimental factors [12]; in other words it is necessary to select genes with the minimum change of quantification cycle (Cq) [13].

The most common genes generally used as internal controls are *β-actin* (*ACTB*), *glyceraldeihyde-3-phospate dehydrogenase* (*GAPDH*) and ribosomal RNAs (rRNAs). Notwithstanding their widespread use, these genes present some drawbacks to keep in mind. For example, *ACTB* is still used despite evidences that its transcription levels, in different cell types, vary widely in response to experimental manipulation as well as during cytoskeletal *de novo* synthesis [14]. In addition, this gene presents many pseudogenes that can lead to misleading results [15]. Also *GAPDH*, which is ubiquitously expressed, was evidenced that its mRNA expression, vary significantly in different circumstances such as during pregnancy, developmental stage, cell cycle and after exposure to different compounds [15]. Furthermore, the promoter of this gene presents multiple insulin-responsive elements and therefore it is stimulated by insulin presence; conversely, in adipocytes, retinoic acid could downregulate *GAPDH* transcription [15]. Regarding rRNAs there are some controversies that affect their selection as RGs: (1) these genes are transcribed by RNA polymerase I and not by polymerase II as is the case of mRNAs, thus their synthesis is independent from each other; (2) these transcripts are not polyadenylated then they cannot be reverse transcribed using oligo(dT) primers; (3) their expression is generally much higher and their degradation is reduced compared to other genes, resulting in too higher amount compared target genes [13].

Consequently, in the last years there are increasing evidences that there are no universal RGs as it is unlikely to identify a single gene whose expression is constant under any treatment [16]. Many researchers suggest to select RGs each time and to validate them under experimental conditions [17]. In order to achieve best results for normalization, it was proposed to follow the common rule of the “Best 3”, which suggests using at least three RGs, three different validation software and three samples (biological replicates) with three technical replicates for each gene [13].

To this aim, the main purpose of this work was to search suitable RGs for qPCR characterization and gene expression analysis on hASCs during long time cultures and to develop an alternative free software to select the proper panel of RGs. We initially considered twelve putative RGs and their stability was evaluated using the three most diffused algorithms (NormFinder, BestKeeper and IdealRef). After that, we selected six genes resulted to be the most stable and investigated their stability in different donors.

## Materials and methods

### hASCs isolation and characterization

hASCs were isolated from mammary adipose tissue of five healthy not smoker women (average age 43±4) who underwent surgery for gigantomasty at the”Ospedale di Circolo”, Varese, Italy. The adipose tissue was obtained after a signed informed consent. All protocols were reviewed and approved by the “University of Insubria” and “Ospedale di Circolo” ethic committees in accordance with the Declaration of Helsinki.

The stromal cellular fraction, obtained according to Gronthos & Zannettino protocol [18], was seeded in flasks and maintained in 15 mL of complete DMEM:DMEM F12 1:1, supplemented with 2–4 mM L-Gln, 1% penicillin-streptomycin, 0.1% gentamicin, 10% FBS, and incubated at 37°C, 5% CO_2_. After 6 hours medium was replaced with fresh medium to remove nonattached cells. Cells were cultured in T75 flasks until eleventh confluence (passage) for a period approximately of three months.

### RNA Extraction and cDNA synthesis

Total RNA was extracted every two passages using the Direct-zol RNA MiniPrep kit (Zymo-Research), according to the manufacturer’s instructions. RNA concentration was determined using Quantus Fluorometer (Promega) and integrity was assessed by gel electrophoresis. The first-strand cDNA was synthesized using the IScript cDNA Synthesis Kit (BioRad) according to the manufacturer’s instructions. At the end of the reaction each sample was diluted 1:10.

### Reference gene selection

Taking into account the most common genes and those most used for mesenchymal stem cells [13,19–22], twelve putative RGs were initially selected (Table 1). To identify the best RGs for gene expression during long culture maintenance, cells were cultivated until the eleventh confluence and collected every two passages. The first analysis was carried on cells extracted from one donor, and then successive studies were conducted on other four patients.

**Table 1:** Putative reference genes selected for the first analysis approach.

Gene	Name	Function
*ACTB*	actin β	Cytoskeleton
*B2M*	β-2-microglobulin	Major histocompatibility complex (MHC) class I heavy chain
*GAPDH*	glyceraldehyde-3-phosphate dehydrogenase	Carbohydrate metabolism
*RPL13A*	ribosomal protein L13a	Component of the 60S subunit
*YWHAZ*	tyrosine 3-monooxygenase/tryptophan 5-monooxygenase activation protein, zeta polypeptide	Signal transduction mediation by binding to phosphoserine-containing proteins
*TBP*	TATA box binding protein	Component of the transcription factor IID
*UBC*	ubiquitin C	Polyubiquitin precursor
*GUSB*	glucuronidase β	Degradation of glycosaminoglycans
*RPS18*	ribosomal protein S18	Component of the 40S subunit
*RPLP0*	ribosomal protein large P0	Component of the 60S subunit
*HPRT1*	hypoxanthine phosphoribosyltransferase 1	Generation of purine nucleotides through the purine salvage pathway
*PPIA*	peptidylprolyl isomerase A (cyclophilin A)	Cyclosporin A-mediated immunosuppression

### Quantitative PCR

qPCR was performed using a CFX96 Connect apparatus (BioRad). The reactions were carried out in triplicates using the SYBR Green PCR super-mix (BioRad), following the manufacturer's instructions. Each reaction was performed on 5 ng of cDNA in a final volume of 10 μL, primers were used at a concentration of 300 nM. Thermocycler program consisted of an initial hot start cycle at 95°C for 3 min, followed by 40 cycles at 95°C for 10 sec and 60°C for 30 sec. To confirm product specificity, a melting curve analysis was performed after each amplification.

The initial panel of genes was analyzed with custom plates preloaded with primers of interest (BioRad). For subsequent experiments, the primer sequences, reported in Table 2, were designed using the software Beacon Designer 7® (BioRad).

**Table 2:** Primer sequences used in this work.

Gene	Primer Sequence (5’ -> 3’)	Amplicon Length (bp)	Accession Number
*ACTB*	Fw: ATGGGTCAGAAGGATTCC Rv: CTCGATGGGGTACTTCAG	78	MN_001101.3
*B2M*	Fw: CTATCCAGCGTACTCCAA Rv: GAAACCCAGACACATAGC	93	NM_004048.2
*GAPDH*	Fw: TTTGGCTACAGCAACAGG Rv: GGTCTCTCTCTTCCTCTTG	107	NM_002046.5
*RPL13A*	Fw: TATGAGTGAAAGGGAGCC Rv: ATGACCAGGTGGAAAGTC	82	NM_012423.3
*RPS18*	Fw: GAGGTGGAACGTGTGATC Rv: GGACCTGGCTGTATTTTC	109	NM_022551.2
*PPIA*	Fw: AACCACCAGATCATTCCTT Rv: GCGAGAGCACAAAGATTC	86	NM_021130.4
*CD44*	Fw: GCAGTCAACAGTCGAAGAAG Rv: GTCCTCCACAGCTCCATT	75	NM_000610.3

### Statistical analysis of reference genes

To define if the panel of RGs was stable, we used the algorithms NormFinder [23], Best Keeper [24] and IdealRef (a personal version of GeNorm) based on the algorithm published by Vandesompele et al. in 2002 [17]. Furthermore, in order to verify IdealRef reliability, results were compared to those obtained by the free software RefFinder [25].

### NormFinder

This approach generates a dimensionless stability value combining intra- and inter-group variation, using log-transformed Cq data. NormFinder needs at least three candidate RGs and a minimum of 8 samples, then it provides a ranking of the evaluated genes, giving a stability value that is low when gene stability is high [19]. The strategy is rooted in a mathematical model of gene expression that enables estimation not only of the overall variation of the genes used for the normalization but also of the variation between sample subgroups. Notably, the strategy provides a direct measure for the estimated expression variation, enabling the user to evaluate the systematic error introduced when using a particular gene [23]. Calculations and advantages over other methods, like the pair wise comparison, are described in details by the authors.

### BestKeeper

The Excel-based tool BestKeeper was developed by Pfaffl and colleagues and it is based on the concept that more stable is the gene expression lower is its Cq variation if the cDNA amount is constant. Cq values are used as input data then the algorithm calculates geometric (Geo Mean), arithmetic mean (Ar Mean) and different values expressing data variation. These values are: minimum and maximum Cq, Cq standard deviation (Std Dev), coefficient of variation (CV); furthermore, the ratio, expressed as x-fold, between minimum and maximum Cq related to the geometric mean and its standard deviation are also provided. Authors listed the genes considering Std Dev and CV and suggested that Cq values with Std Dev higher than 1 are considered inconsistent. They also suggested to use more than one RG in order to get more reliable results [19]. Moreover, the algorithm performs pair-wise correlation analyses to estimate inter-gene relations of all possible RG pairs. For every calculated correlation, the Pearson correlation coefficient (r) and the probability p value are calculated. All those highly correlated House Keeping Genes (HKGs) are combined into an index, and then the correlation between each candidate HKG and the index is described by the Pearson correlation coefficient (r), coefficient of determination (r2) and the p-value [24].

### IdealRef

Starting from the published algorithm, we developed our personal version that is freely downloadable from the personal website of the developer at www.pela.it. Our software uses raw Cq values as input and need to select a target gene. The software calculates the average Cq samples and then it gives expression of the target gene (2^-ΔCq^) normalized with one RG at a time. From these values, the algorithm produces a dimensionless value (GS value) indicating the stability of the RG. This algorithm is based on the principle that the expression ratio of two ideal control genes is identical in all samples, regardless of the experimental condition [17]. Following a stepwise exclusion, genes can be ranked from the most instable (higher GS value) to the most stable (lower GS value). Genes are considered stably expressed when GS value is below 0.5 if the sample is homogeneous, *i*.*e*. cell culture of the same cell type [19].

Moreover, the algorithm calculates the minimum number of RGs required for a correct normalization without insert instable genes in the gene set to use. This parameter, named NG value, is the ratio between the first three stable genes and the stepwise inclusion of the subsequent stable gene according to GS value and it is repeated until all the genes are inserted. Authors of GeNorm software have fixed the cut-off of NG value at 0.15, below which the inclusion of an additional control gene is not required [17].

### RefFinder

This user-friendly web-based comprehensive tool was developed for evaluating and screening RGs from extensive experimental datasets. It integrates the currently available major computational programs (GeNorm, Normfinder, BestKeeper, and the comparative ΔΔCt method) to compare and rank the tested candidate RGs. Based on the rankings from each software, it assigns an appropriate weight to an individual gene and calculates the geometric mean of their weights for the overall final ranking [25].

## Results

### hASCs characterization

After extraction from adipose tissue of healthy donor, hASCs were characterized by flow cytometry and the results have been reported in a previous paper [26].

### First analysis of reference genes initially selected

After qPCR, Cq values were used to evaluate stability of RGs by the three algorithms mentioned above. Among the genes reported in Table 1, *TBP*, *GUSB* and *HPRT1*, were not taken into account for subsequent studies as their average Cq was higher than 24. The remaining genes were analyzed and the results obtained from NormFinder, BestKeeper and IdealRef are reported as Tables.

NormFinder algorithm assigns a lower value for higher stable gene and indicates that, in our experimental conditions, the three most stable genes are *RPL13A*, *RPS18* and *GAPDH*. Standard errors of these genes are very low, indicating that small error is introduced when a researcher uses these genes as reference (Table 3).

**Table 3:** Analysis of the investigated genes by NormFinder algorithm.

Order of gene stability	Stability value	Standard error
*RPL13A*	0.104	0.067
*RPS18*	0.155	0.067
*GAPDH*	0.166	0.068
*YWHAZ*	0.212	0.075
*B2M*	0.238	0.080
*UBC*	0.310	0.095
*PPIA*	0.470	0.132
*RPLP0*	0.488	0.137
*ACTB*	0.498	0.139

BestKeeper criterion to choose the best RGs relies on the principle that more a gene is stable lower is its Cq variation. After the software calculated descriptive statistics for the individual candidate, the user can select the most stable genes on the base of standard deviation values. Our data indicates that the most stable genes are *RPL13A*, *ACTB* and *RPS18*. No gene shows Std Dev higher than 1, consequently all genes can be considered enough stable (Table 4).

**Table 4:** Analysis of the investigated genes by BestKeeper algorithm.

	*RPL13A*	*ACTB*	*RPS18*	*YWHAZ*	*B2M*	*PPIA*	*GAPDH*	*UBC*	*RPLP0*
Geo Mean [Cq]	20.58	20.58	19.51	21.95	18.94	19.52	18.79	21.07	20.80
Ar Mean [Cq]	20.58	20.58	19.52	21.95	18.95	19.53	18.80	21.08	20.80
min [Cq]	20.19	20.18	18.80	21.26	18.39	18.71	18.00	20.47	20.06
max [Cq]	21.19	21.17	20.19	22.86	19.83	20.61	19.81	22.09	22.02
Std Dev [±Cq]	0.27	0.32	0.35	0.38	0.38	0.39	0.40	0.42	0.45
CV [% Cq]	1.33	1.56	1.77	1.71	2.02	2.01	2.14	1.99	2.14
min [x-fold]	-1.31	-1.32	-1.64	-1.61	-1.47	-1.76	-1.72	-1.52	-1.66
max [x-fold]	1.53	1.51	1.60	1.88	1.85	2.14	2.02	2.02	2.33
Std Dev [±x-fold]	1.21	1.25	1.27	1.30	1.30	1.31	1.32	1.34	1.36

Furthermore, BestKeeper software produces pair-wise correlation results in order to estimate the relationship among reference gene pairs. Pearson correlation coefficient (r), coefficient of determination (r^2^) and the p-value between the bestkeeper gene and the candidate gene are reported in Table 5. Among the candidate genes three of them, *ACTB*, *PPIA* and *RPLP0*, have a p-value higher than 0.05, indicating that they are not correlated with the BestKeeper index and therefore they are not good RGs. Good candidate RGs are indicated in bold.

**Table 5:** BestKeeper pair-wise correlation.

	*RPL13A*	*ACTB*	*RPS18*	*YWHAZ*	*B2M*	*PPIA*	*GAPDH*	*UBC*	*RPLP0*
coeff. of corr. [r]	0.97	0.32	0.93	0.92	0.89	0.67	0.97	0.83	0.69
coeff. of det. [r^2^]	0.94	0.10	0.87	0.84	0.80	0.44	0.94	0.69	0.48
p-value	**0.001**	0.438	**0.001**	**0.001**	**0.003**	0.072	**0.001**	**0.011**	0.056

To experiment the validity of IdealRef software we selected *CD44* as target gene since it is one the most important surface marker to characterize hASCs [9]. According to IdealRef software, high stable genes show low GS values, calculated as the average pairwise variation of a particular gene compared to the other control genes. Results, reported in Table 6, indicate that the most stable genes are *RPS18* and *RPL13A* (0.207) and *GAPDH* (0.235). For homogeneous samples, as in our case, good RGs must have GS values lower than 0.5 consequently *RPLP0* and *ACTB* should be excluded. In addition, the minimum number of genes required for gene expression normalization is calculated adding step by step the gene with increasing instability. NG values, summarized in Table 7, indicate that the best combination of genes is *RPS18*, *RPL13A* and *GAPDH*. The cut off proposed by Vandesompele and coworkers is 0.15 and all values obtained from our experiments are below this value, then three RGs are enough for normalization procedure.

**Table 6:** GS value resulted from IdealRef.

	GS value
*RPS18—RPL13A*	0.207
*GAPDH*	0.235
*YWHAZ*	0.258
*B2M*	0.275
*UBC*	0.369
*PPIA*	0.458
RPLP0	0.527
ACTB	0.576

**Table 7:** NG values calculated by IdealRef.

Number of genes	NG value
**3/4**	0.016
**4/5**	0.014
**5/6**	0.013
**6/7**	0.013
**7/8**	0.014
**8/9**	0.015

In order to confirm the results produced by IdealRef and to summarize the gene list obtained from all algorithms, raw Cq data were analyzed also by RefFinder software (Table 8). This analysis produced the same ranking that we have previously obtained with IdealRef, confirming that our algorithm works properly. Moreover, it provides a comprehensive ranking that takes into account the three algorithms.

**Table 8:** Integrated results produced by RefFinder.

	Normfinder	BestKeeper	Genorm	Recommended comprehensive ranking
**1**	*RPL13A*	*RPL13A*	*RPL13A | RPS18*	*RPL13A*
**2**	*RPS18*	*ACTB*		*RPS18*
**3**	*GAPDH*	*RPS18*	*GAPDH*	*GAPDH*
**4**	*YWHAZ*	*YWHAZ*	*YWHAZ*	*YWHAZ*
**5**	*B2M*	*B2M*	*B2M*	*B2M*
**6**	*UBC*	*PPIA*	*UBC*	*ACTB*
**7**	*PPIA*	*GAPDH*	*PPIA*	*UBC*
**8**	*RPLP0*	*UBC*	*RPLP0*	*PPIA*
**9**	*ACTB*	*RPLP0*	*ACTB*	*RPLP0*

### Analysis of selected reference genes in different patient

Once selected suitable RGs, we verified the gene stability in four donors.

In general, in qPCR experiments, it is recommended to use between two and five validated stably expressed RGs (https://www.qbaseplus.com/knowledge/blog/four-tips-rt-qpcr-data-normalization-using-reference-genes?page=1). In order to analyze whether the RG stability was donor-dependent and considering the results obtained from all the algorithms, we selected the six most stable genes (Table 8). Although *YWHAZ* resulted to be more stable than *PPIA*, we chosen this one as its average Cq values are in the same range of the other five genes. Analysis was performed independently from each other and the results are summarized in Table 9.

**Table 9:** Ranking obtained by all the three software.

**Donor 2**	**NormFinder**	**BestKeeper**	**IdealRef**	**RefFinder**
1	*RPS18*	*B2M*	*RPS18—B2M*	*RPS18*
2	*GAPDH*	*RPS18*		*B2M*
3	*B2M*	*PPIA*	*RPL13A*	*GAPDH*
4	*RPL13A*	*RPL13A*	*GAPDH*	*RPL13A*
5	*PPIA*	*GAPDH*	*PPIA*	*PPIA*
6	*ACTB*	*ACTB*	*ACTB*	*ACTB*
**Donor 3**	**NormFinder**	**BestKeeper**	**IdealRef**	**RefFinder**
**1**	*GAPDH*	*RPS18*	*RPL13A - GAPDH*	*GAPDH*
**2**	*RPL13A*	*PPIA*		*RPL13A*
**3**	*PPIA*	*GAPDH*	*B2M*	*PPIA*
**4**	*RPS18*	*RPL13A*	*PPIA*	*RPS18*
**5**	*B2M*	*ACTB*	*RPS18*	*B2M*
**6**	*ACTB*	*B2M*	*ACTB*	*ACTB*
**Donor 4**	**NormFinder**	**BestKeeper**	**IdealRef**	**RefFinder**
**1**	*RPS18*	*RPL13A*	*RPS18—B2M*	*RPS18*
**2**	*B2M*	*GAPDH*		*B2M*
**3**	*GAPDH*	*RPS18*	*RPL13A*	*RPL13A*
**4**	*RPL13A*	*B2M*	*GAPDH*	*GAPDH*
**5**	*PPIA*	*ACTB*	*PPIA*	*PPIA*
**6**	*ACTB*	*PPIA*	*ACTB*	*ACTB*
**Donor 5**	**NormFinder**	**BestKeeper**	**IdealRef**	**RefFinder**
**1**	*GAPDH*	*GAPDH*	*RPS18—RPL13A*	*PPIA*
**2**	*PPIA*	*PPIA*		*GAPDH*
**3**	*RPL13A*	*B2M*	*B2M*	*RPL13A*
**4**	*B2M*	*RPL13A*	*PPIA*	*RPS18*
**5**	*RPS18*	*RPS18*	*GAPDH*	*B2M*
**6**	*ACTB*	*ACTB*	*ACTB*	*ACTB*

For each donor, RefFinder confirmed the ranking obtained by IdealRef. Even though the list of RGs is different for each of the considered patient, NG values, calculated by IdealRef, indicate that in all cases three genes are enough for normalization (Table 10).

**Table 10:** NG values calculated by IdealRef for each donor.

Number of genes	Donor 2	Donor 3	Donor 4	Donor 5
**3/4**	0.033	0.033	0.039	0.022
**4/5**	0.024	0.025	0.030	0.017
**5/6**	0.024	0.025	0.028	0.013

## Discussion

Despite hASCs are being increasingly important in the field of reconstructive medicine there is a lack of standardized procedure for their characterization. qPCR is a good choice as it is simple to perform and cheaper compared to other techniques. In this context, the absolute prerequisite to correctly measure gene expression is the accurate normalization resulting in the selection of RGs whose expression is stable under experimental conditions. In 2000 Schmittgen and Zakrajsek published the first article about the need to validate RGs [16] and, in recent years, it has become clear that no single gene is equally expressed in all cell types and in different experimental conditions. This assumption implies that, when relative quantification is used, the stability of RGs has to be verified before each experiment [17]. Unfortunately, the importance of selecting appropriate RGs is often overlooked and then mRNA expression normalization, conducted using considering genes whose expression that fluctuate depending on experimental conditions can have profound effects on data analysis [27].

While it seems unlikely that we can identify a gene whose expression is constant, it is possible to select RGs that are stable under a subset of selected conditions [28]. Moreover, there is no *a priori* reason to expect that the same RG will be stable across different tissues, or even cell lines from the same tissue type [27].

With this work, we have developed a new free software “IdealRef” to select a panel of proper RGs constantly expressed in hASCs during long time culture in order to characterize them for future studies. The step by step analysis using three software allowed us to identify six stable genes (*ACTB*, *B2M*, *GAPDH*, *RPL13A*, *RPS18*, *PPIA*) whose stability was analyzed in four donors. Despite its widespread use, *ACTB*, as calculated by IdealRef, resulted to be the worst RG in all considered patients, and our outcome, confirmed by other researchers [29–32], discourage its use as RG. In fact, this gene is proved to be unstable also in bone marrow-derived mesenchymal stem cells and fetal tissue-derived mesenchymal stem cells [29]. During *in vitro* maintenance, stem cells are subjected to mechanical forces that regulate cellular responses to microenvironment that involve cytoskeletal organization of which *ACTB* is an important constituent. However, the exact intracellular mechanisms that govern these responses in stem cells have yet to be fully characterized, making genes involved in cytoskeletal remodeling unsuitable as RGs [14].

Debated is also the use of *GAPDH*, in fact, in one study on adipose tissue biopsies, 18S rRNA and *GAPDH* are found to be respectively the most and the least suitable RG [28]. Conversely, in other studies on cultured adipocytes and preadipocytes, exposed to different hormones, as well as in pluripotent stem cells, the authors demonstrated that *GAPDH* could be a good RG [13,33]. *GAPDH*, besides being involved in cytoskeletal organization [34], catalyzes an important energy-yielding step in carbohydrate metabolism, and studies of *GAPDH* in mouse have assigned to this gene a variety of additional functions. Considering its involvement in so many processes, taking into account the data from literature and the results of our study that indicates that *GAPDH* occupies discordant positions in the examined patients, it is clear that is absolutely necessary to validate *GAPDH* each time.

The two ribosomal proteins *RPS18* and *RPL13A* maintained relative stability of expression across organ/tissue types [21,22] and our analysis supports this finding. Our results is also in agreement with literature reporting that *RPLP0* expression is tissue dependent [21]; in fact the first analysis we conducted in our samples evidenced that *RPLP0* is not stable enough, then it was excluded from successive analyses. Not surprisingly, the three models used gave different stability rankings and the reason could be that they are based on different assumptions. Therefore, it should always be a priority to find genes that are considered good references according to different algorithms [13].

Our data highlight the great variability among donors even presenting the same characteristics; this reinforces the idea that it is highly unlikely that universal RGs can exist [35]. In fact, a gene validated in particular conditions by a laboratory may not fit as well for the conditions and samples used in other laboratories. It is clear that it is not possible to predict which housekeeping gene may be useful [12] and that is why an important step is the selection of genes whose expression stability has to be verified before conducting the experiment [13]. Considering also the variability within samples, this work highlights that is important to follow such kind of approach in order to correctly conduct RG selection and hence reach more precise results when using qPCR.

Despite all these demonstrations, many researchers continue to use a single, invalidated RG to normalize their data [27]. To our knowledge, this is the first study that evaluates differences in gene expression of hASCs extracted from different patients during long culture maintenance. It is important to publish such studies in order to provide a useful starting point to identify which RGs may be appropriate to include in a test panel for one’s own experimental set-up as well as primer sequences and methodological details that can be invaluable to others.
